# Reference intervals of complete blood count parameters in the adult western Sudanese population

**DOI:** 10.1186/s13104-024-06754-3

**Published:** 2024-04-02

**Authors:** Malak Ibrahim Mustafa, Ibrahim Abdelrhim Ali, Muaath Ahmed Mohammed, Elmutaz Hussien Taha, Kamal Mohamed Awad, Omer Abdelaziz Musa

**Affiliations:** 1 Department of Physiology, Faculty of Medicine, The National Ribat University, Khartoum, Sudan; 2 Department of Physiology, Faculty of Medicine, University of Dongola, Dongola, Sudan; 3 Department of Physiology, Faculty of Medicine, University of Gadarif, Elgadarif, Sudan

**Keywords:** Parametric method, Reference intervals, CBC, HGB, RBCs, WBCs, PLT

## Abstract

**Background:**

A complete blood count (CBC) analysis is one of the most common conventional blood tests that physicians frequently prescribe.

**The objective:**

of this study was to determine the reference intervals (RIs) of CBC parameters in the population of healthy adults living in the western Sudan region.

**Methods:**

A cross-sectional study of healthy people residing in the western area of Sudan was carried out. We assessed the CBC RIs in samples taken from 153 individuals using an automated haematology analyser (Sysmex KX-21) and a modified Box–Cox transformation procedure to transform the data into a Gaussian distribution after eliminating outliers using the Dixon method. IBM SPSS Statistics version 25 was used to analyse the data, and t tests were employed to examine variations in the mean CBC parameters according to sex and age. P was considered significant at ≤ 0.05.

**Results:**

Beyond all the other measured values, the only CBC parameters that significantly differed between the sexes were haemoglobin (HGB) and white blood cell (WBC) counts. Women were found to experience more WBC counts than men did. However, they have less HGB RIs.The male participants in our study exhibited lower WBC count RIs, a significantly lower limit, and a greater upper limit of platelet RIs than did the individuals from other nations.

**Conclusions:**

Compared with males, females had higher platelet and WBC counts and lower HGB.

## Introduction

A complete blood count (CBC) is an essential tool for the diagnosis and treatment of hematologic illnesses as well as for the general assessment of an individual’s health [1]. However, recent research has shown that there are notable variations in hematological reference values between various populations, underscoring the significance of creating reference intervals (RIs) unique to each ethnic group [2, 3]. Inappropriate RIs can result in incorrect patient care and misclassification; hence, RIs that take geography, race, and ethnicity into consideration are essential for screening, diagnosing, treating, and monitoring hematological disorders [4].To guarantee correct evaluations, the International Federation of Clinical Chemistry (IFCC) and the Clinical and Laboratory Standards Institute (CLSI) advise laboratories to develop their own RIs [5–7].

An essential measure of immunological response and inflammation is the white blood cell (WBC) count. Age, sex, and ethnicity are a few variables that may have an impact on variations in WBC count [8, 9]. For instance, African Americans typically have higher WBC counts than Caucasian people [10]. It is important to take these differences into account when assessing WBC counts in various groups.

Factors, including ethnicity, smoking status, and altitude, can all have an impact on variations in red blood cell (RBC) counts [11, 12]. People who live at high elevations, for instance, might have increased RBC counts because of the body’s adaptation to low oxygen levels [12]. Similarly, research has revealed that people of African heritage typically have greater RBC counts than people from other groups [13].A number of variables, including age, sex, altitude, and ethnicity, might affect haemoglobin (HGB) levels [14]. For instance, research indicates that because of the body’s adaptation to low oxygen levels, individuals who live at high elevations typically have lower haemoglobin levels [15]. Furthermore, differences in haemoglobin levels have been noted among several ethnic groups [16–24].

Age, sex, and ethnicity are a few variables that may have an impact on platelet count [25]. For instance, research indicates that men often have larger platelet counts than women [25]. Furthermore, differences in platelet counts have been noted between several ethnic groups [16–24].

The variation in the size of red blood cells was measured by determining the red cell distribution width (RDW). Research has indicated that older people typically have greater RDWs than younger people [26]. Furthermore, differences in RDW have been noted among several ethnic groups [16–24].

Since each population is unique and several factors can influence normal physiological values, it is imperative to obtain reference values from the local population before applying them in a clinical setting [27]. In Sudan, where blood cell reference values have not yet been established, it is common to rely on values from developed countries. Therefore, this study aimed to determine the reference intervals for CBC parameters in a healthy adult population residing in western Sudan, focusing on the specific variations observed within this population.

## Methods

### Study design, duration and setting

A cross-sectional descriptive study of healthy adults residing in Al Fashir city, the capital of the western Sudan region (which includes Darfur and Kordofan), was carried out in September and October 2019. At the time of the most recent census, the region’s estimated population was 14.448.738. Its area is 869.325 km² (337.352 miles²).

### Study population and eligibility criteria

Study participants were chosen on the basis of their clinical history, physical examination, and CLSI-IFCC inclusion and exclusion criteria [5]. The inclusion criteria for individuals were as follows: male or female between the ages of 18 and 64, inhabited by western Sudan for at least six months, and seronegative for hepatitis B and C viruses. The exclusion criterion included having any medical condition, such as high blood pressure, anaemia, or Cardio Vascular Diseases (CVD). Drug treatment, alcohol use, heavy smoking, chronic illnesses such as diabetes mellitus (DM), and blood donation within the previous three months were additional exclusion criteria. People who had recently undergone surgery were also excluded.

### Sample size and technique

To establish reference intervals using the percentile method, the IFCC-CLSI recommends a sample size of 120 samples for each stratum [5]. For robust and parametric methods, the recommended sample size is at least 40 samples per category. In this study, we used a parametric method to establish RIs among 153 subjects.

### Data collection and study procedure

Each of the chosen participants completed a questionnaire with detailed personal information, medical history, level of physical activity, food, and smoking status. To analyse 18 complete blood count (CBC) parameters (white blood cell (WBC), red blood cell (RBC), platelet (PLT), haemoglobin (HGB), haematocrit (HCT), mean corpuscular voulume (MCV), mean corpuscular haemoglobin (MCH), mean corpuscular haemoglobin concentration (MCHC), red cell distribution width count and percentage (RDW), platelet distribution width (PDW), mean platelet volume (MPV), leukocyte differential absolute counts (abs) and percentages (%) of neutrophils (Neu), lymphocytes (Lym) and mixed (monocyte (Mon), eosinophil (Eos), basophil (Bas)) cells (mxd)), a sample of 2–3 ml of venous blood was drawn and placed into vacutainer tubes containing ethylenediaminetetraacetic acid (EDTA) anticoagulant. The time frame for collecting the samples was 8:00 a.m. to 11 a.m. Following the blood and anticoagulant mixture, the test vials were properly labelled. After that, the samples were placed in an ice box, brought to the laboratory, and processed for two hours. A Sysmex KX-21 automated haematology analyser at Alfashir Police Hospital Laboratory was used to analyse the blood.

### Quality control

Precautions for IFCC-CLSI sample analysis were taken into account. Every day, the analyser was calibrated using industry standards. 10% of the samples were reanalyzed in the reference laboratory as part of an external quality control measure.

### Data analysis

SPSS version 25, or the Statistical Package for the Social Sciences, was used to conduct the statistical analysis. The data distribution was assessed using the Shapiro‒Wilk and Kolmogorov‒Smirnov tests. A modified Box–Cox transformation formula was used to convert the data into a Gaussian distribution so that reference intervals could be calculated using a parametric approach. Using the Dixon method, the participants were examined for outliers. The data included the mean, standard deviation, and reference intervals (mean ± 1.96*SD), which represent the central 95% lower (LL) and upper limits (UL) under the transformed scale. The LL and UL at the original scale are then obtained by reversing the limits. In addition, the obtained reference intervals were contrasted with values from other African nations. Student’s t test was used to assess the relationships between the participants’ age and sex and blood parameters. A P value ≤ 0.05 was considered to indicate statistical significance.

## Results

The purpose of the study was to determine the reference values for complete blood counts in adults in western Sudan who were in good health.

### The participants’ characteristics

In all, 172 participants in the 18–64 age range participated in the study. Nineteen patients were eliminated owing to diabetes mellitus (3), hypertension (5), high BMI (3), or outliers (8), resulting in a final sample size of 153. There were 56 (36.7%) males and 97 (63.3%) females in total, with a mean age of 23.5 years.

### Determination of reference intervals

Reference intervals (means ± 1.96*SD) were obtained for each of the eighteen complete blood count parameters through the parametric approach. Table 1. Of the complete blood count parameters measured, only WBC and HGB counts significantly differed between the sexes. Table 2. Age was not a significant (p value ˃ 0.05) source of variation for any of the measured CBC parameters.

**Table 1:** Reference intervals for the participants’ CBC parameters that were obtained parametrically

	Sex							
	Male				Female			
	Mean	SD	L.L	U.L	Mean	SD	L.L	U.L
WBCs *103/ul	5.28	1.40	2.5	8.0	6.01	1.59	2.8	9.1
RBCs*106/ul	5.48	0.43	4.6	6.3	4.88	0.38	4.1	5.6
HGB g/dl	15.70	1.52	12.7	18.6	13.38	1.48	10.4	16.2
HCT %	49.73	4.06	41.8	57.5	43.54	5.72	32.3	54.7
MCV (fl)	91.34	4.93	81.6	101	89.24	6.76	75.9	102.4
MCH (pg)	28.73	2.20	24.4	33	27.45	2.83	21.9	32.9
MCHC g/dl	31.47	0.95	29.6	33	30.74	1.09	28.7	32.7
PLT* 103/ul	232.42	74.99	85.4	379	312.64	88.21	139.7	485.5
LYM%	44.34	8.91	26.8	61.8	42.76	8.61	25.8	59.6
MXD%	11.04	2.28	6.5	15.4	10.13	2.65	4.9	15.3
NEUT%	44.63	9.08	26.9	62	47.11	9.09	29.4	64.7
LYM 103/ul	2.28	0.59	1.12	3.4	2.51	0.59	1.3	3.6
MXD 103/ul	0.57	0.18	0.217	0.922	0.58	0.20	0.18	0.97
NEUT 103/ul	2.42	1.12	0.224	4.6	2.92	1.29	0.39	5.4
RDW_SD (fl)	48.51	4.31	40	56.9	50.08	3.74	42.6	57.3
RDW_CV%	13.80	0.93	11.9	15.6	14.84	2.68	9.5	20
PDW(fl)	15.80	0.44	14.9	16.6	15.53	0.36	14.8	16.2
MPV(fl)	8.74	0.55	7.6	9.8	8.78	0.66	7.4	10

**Table 2:** Relationships between age, sex and blood parameters, (WBC and HGB)

		Mean	Standard deviation	Reference intervals	P value
**WBC**					
Sex	Male	5.28	1.40	2.5–8	0.003
	Female	6.01	1.59	2.8–9	
**HB**					
Sex	Male	15.70	1.52	12.7–18.6	0.000
	Female	13.38	1.48	10.4–16.2	

T test results A P value ≤ 0.05 was considered to indicate statistical significance

The reference intervals determined in this study were also contrasted with those reported in related studies conducted in Kenya (18) and Eritrea (19) using parametric methods and nonparametric methods, respectively. Table 3.

**Table 3:** Comparing the results of our research with those published in other countries

Parameter	Sex	Our values	Kenya (18)	Eritrea (19)
WBCs *103/ul	Male Female	2.5-8.0 2.8–9.1	3.1–8.1 2.8–7.7	3.7–9.3 3.3–8.9
RBCs*106/ul	Male Female	4.6–6.3 4.1–5.6	4.94–6.52 4.31–5.76	4.2–6.07 4–5.7
HGB g/dl	Male Female	12.7–18.6 10.4–16.2	14.5–18.7 12.0–16.5	12.6–17.8 12.5–17.6
HCT %	Male Female	41.8–57.5 32.3–54.7	**-** **-**	40.5–55 37.9–52
MCV (fl)	Male Female	81.6–101 75.9-102.4	76.5–95.5 73.4–95.8	85.7–100 85.5–100
MCH (pg)	male female	24.4–33 21.9–32.9	25.1–32.8 24.4–32.7	28–33 26.5–32.6
MCHC g/dl	male female	29.6–33 28.7–32.7	32.4–35.4 32.0–35.0	30.4–33.7 30–33.7
PLT* 103/ul	male female	85.4–379 139.7-485.5	133–356 152–443	128.4–318.4 145.4–351.6
LYM%	male female	26.8–61.8 25.8–59.6	28.2–60.3 25.5–59.3	**-** **-**
NEUT%	male female	26.9–62 29.4–64.7	27.4–60.3 29.5–65.4	**-** **-**
LYM 103/ul	male female	1.12–3.4 1.3–3.6	1.36–3.58 1.22–3.24	**-** **-**
NEUT 103/ul	male female	0.224-4.6 0.39–5.4	1.02–3.92 1.07–4.42	
RDW_CV%	male female	11.9–15.6 9.5–20	**-** **-**	12.3–15.5 12.3–17

## Discussion

The complete blood count (CBC) test is essential for diagnosing a wide range of illnesses and assessing overall health. The outcomes were influenced by a number of factors, such as geography and sex. The purpose of this study was to ascertain CBC reference intervals for the population of western Sudan region to determine the standards for laboratory results. The study’s findings highlight the fact that the RIs discovered in one community should not be extrapolated to other communities.

According to our research, the RIs for several RBC parameters, such as RBC count, HCT, MCV, MCH, MCHC, and RDW, did not differ according to sex. However, males had a greater HGB than females did. This result is consistent with earlier research conducted in African nations [20, 21]. A poor diet; frequent childbirth; monthly blood loss in women; and the effects of androgen, oestrogens, and testosterone on RBC synthesis are thought to be the main causes of these sex differences [14, 18, 23, 28]. Furthermore, a World Health Organization (WHO) report [29] states that a woman over the age of 15 is considered anaemic if her HGB is less than 12 g/dl. Therefore, given that we report a lower limit (10.4 g/dl) for HGB reference intervals in our study, a certain percentage of women are anemic.

There was a noticeable difference in the RI of the total WBC count between males and females. These results are consistent with research conducted in Turkey [30]. This contradicts the results of a study conducted in the Central African Republic [31]. Although there was considerable sex variation in the WBC count, the WBC differential (LYM, NEUT, and MXD) counts and percentages of males and females did not exhibit any discernible sex variation. These results are consistent with those of other studies conducted in the Central African Republic and Turkey [30, 31]. In addition, our results imply that, in comparison to those of the Eritrean study, the male WBC count reference intervals were lower [19].

Additionally, there was no statistically significant difference in platelet count or platelet indices (PDW or MPV) between the sexes according to our study. These findings are consistent with those of other research carried out in Ghana and Kenya [24, 32]. According to our research, the PLT reference intervals in males had a greater upper limit and a significantly lower limit. This finding is in contrast to research conducted in Kenya and Eritrea [18, 19]. Although our investigation of reduced platelet count is in line with findings from other countries [28, 32], the reason for this difference has yet to be determined.

The parametric method is more dependable than the nonparametric method at driving precise and narrower reference intervals because it includes a step for excluding values outside of the mean, can successfully transform data into a normal distribution shape by using a modified Box–Cox transformation formula, and provides a fairly consistent optimal power value for the Gaussian transformation parameter by parameter [27]. These facts are established unequivocally by comparing the reference intervals generated by the two techniques in the Kenyan study [18].

## Conclusion

The HGB and WBC counts were significantly different according to sex, with women having lower HGB and higher WBC count RIs than men. Compared to that in previous studies, the lower limit of the platelet count RIs in males was significantly lower.

### Strengths and limitations

To the best of our knowledge, this is the first study to create CBC RIs for adults living in western Sudan; the created RIs might be useful for diagnostic laboratories in the area. Additionally, RIs obtained using the parametric methods are more accurate than those derived using other methods. However, there was a drawback to our research. The recruitment of samples and the dominance of particular tribes in the area may introduce selection bias, which could restrict the ability to generalize the findings.

## Data Availability

The corresponding author can provide the datasets used and analysed for this study upon reasonable request.
